# Cardiac magnetic resonance based deformation imaging: role of feature tracking in athletes with suspected arrhythmogenic right ventricular cardiomyopathy

**DOI:** 10.1007/s10554-018-1478-y

**Published:** 2018-10-31

**Authors:** Csilla Czimbalmos, Ibolya Csecs, Zsofia Dohy, Attila Toth, Ferenc Imre Suhai, Andreas Müssigbrodt, Orsolya Kiss, Laszlo Geller, Bela Merkely, Hajnalka Vago

**Affiliations:** 1 Heart and Vascular Center, Semmelweis University, Budapest, Hungary; 2 Department of Electrophysiology, Heart Centre, University of Leipzig, Leipzig, Germany

**Keywords:** Arrhythmogenic right ventricular cardiomyopathy, Athlete’s heart, Cardiac magnetic resonance imaging, Feature tracking

## Abstract

Both, arrhythmogenic right ventricular cardiomyopathy (ARVC) and regular training are associated with right ventricular (RV) remodelling. Cardiac magnetic resonance (CMR) is given an important role in the diagnosis of ARVC in current task force criteria (TFC), however, they contain no cut-off values for athletes. We aimed to confirm the added value of feature tracking and to provide new cut-off values to differentiate between ARVC and athlete’s heart. Healthy athletes with training of minimal 15 h/week (n = 34), patients with definite ARVC (n = 34) and highly trained athletes with ARVC (n = 8) were examined by CMR. Left and right ventricular volumes and masses were determined. Global right and left ventricular, and regional strain analysis for the RV free wall was performed using feature tracking on balanced steady-state free precession cine images. 94% of healthy athletes showed RV dilatation of the proposed TFC, 14.7% showed RV ejection fraction (RVEF) between 45–50%, none of them had RVEF < 45%. Although RVEF showed the highest accuracy in differentiating between athlete’s heart and ARVC, only 37.5% of athletes with ARVC showed RVEF < 45%. The only parameters falling in the pathological range (based on our established cut-off values: > − 25.6 and > − 1.4, respectively) in all athletes with ARVC were the strain and strain rate of the midventricular RV free wall. Establishing RVEF and RV strain analysis provides an important tool to distinguish ARVC from athlete’s heart. CMR based regional strain and strain rate values may help to identify ARVC even in highly trained athletes with preserved RVEF.

## Introduction

Prolonged endurance exercise may lead to pronounced morphological changes of the right ventricle [1]. However, certain exercise induced alterations seem to be a benign consequence of athletic performance, occasionally observed overlap between arrhythmogenic right ventricular cardiomyopathy (ARVC) and physiological right ventricular adaptation may lead to clinical challenges. As in some European countries up to 20% of sudden cardiac deaths (SCD) in young individuals and athletes may be caused by ARVC [2, 3], thus differentiation between physiological and pathological RV remodelling is essential as correct diagnosis may prevent SCD. Moreover, misdiagnosis of ARVC may have serious health- and career-related consequences.

Overlapping electrical and structural features of athlete’s heart and ARVC—such as right bundle branch block, T-wave inversion in precordial leads, elevated RV end-diastolic volume (RVEDVi) or slightly decreased EF—may cause diagnostic difficulties. Current TFC require as a major criterion regional RV akinesia or dyskinesia or dyssynchronous RV contraction and RVEDVi ≥ 110 ml/m^2^ (male) or ≥ 100 ml/m^2^ (female) or RV ejection fraction ≤ 40%. As a minor criterion regional RV akinesia or dyskinesia or dyssynchronous RV contraction and a ratio of RVEDVi ≥ 100 to < 110 ml/m^2^ (male) or ≥ 90 to < 100 ml/m^2^ (female) or RV ejection fraction > 40% to ≤ 45% is required [4]. Thus, morphological features of ARVC require wall motion abnormalities and increased RV volume or decreased RV ejection fraction. As the diagnosis of wall motion abnormalities is based on individual subjective judgement and current TFC contain no athlete-specific criteria adapted for the objective parameters RVEDVi and RVEF [4], misdiagnosis of ARVC in healthy athletes may occur frequently. Therefore, improved cut-off values for CMR parameters differentiating ARVC and athlete’s heart are needed.

Tissue tracking technology such as speckle tracking echocardiography and feature tracking CMR recently have become a subject of great interest for clinicians managing patients with ARVC. Feature tracking technique allows us to estimate myocardial strain, a measure of deformation using balanced steady-state free precession (bSSFP) cine images. Recent literature data imply, that CMR based deformation imaging using feature tracking technology may have an important added value in the diagnostic workup of ARVC patients [5–8], although no data regarding athletes’ normal RV values are available. To the best of our knowledge, this is the first study to compare healthy athletes’ and ARVC patients’ strain parameters using feature tracking.

Connection between sport activity and ARVC is an intensively studied topic. It is a proven fact, that high intensity exercise is a strong independent marker of life-threatening arrhythmias [9] and exercise dose reduction results in decreased risk of ventricular arrhythmias [10], therefore competitive sport is discouraged in ARVC patients [11]. Although CMR is the gold standard non-invasive method to measure RV volumes and function, there are only few studies investigating the role of athletic performance using CMR [12–14]. Not surprisingly, competitive athletes have significantly larger RV volumes than recreational athletes or sedentary patients. In terms of RVEF literature data are contradictory. Saberniak et al. showed that athletes have reduced ejection fraction compared to non-athletes, suggesting that sport accelerates ventricular dysfunction in ARVC, while Ruwald et al. could not prove any difference in EF between competitive athletes, recreational athletes and inactive patients. Unfortunately these studies aimed to analyse only RV volumes and ejection fraction, more detailed CMR characteristics (including LGE or RV deformation imaging) of competitive athletes are reported only in case reports, or series [15–17].

Our goal was to compare conventional left and right ventricular parameters of healthy athletes with non-athletic ARVC patients, and to investigate clinical and detailed CMR characteristics of active highly trained athletes with ARVC. We tested the diagnostic accuracy of TF CMR criteria regarding RVEDVi and RVEF, and we tested whether global or regional strain analysis could improve the diagnostic value of CMR in this special patient population.

## Materials and methods

### Study participants

This study was conducted between 2010 and 2017. ARVC patients with definite diagnosis based on the revised Task Force criteria were consecutively enrolled (n = 34). Highly trained healthy athletes with a minimum of 15 h of training per week for at least 5 years performing sports with high dynamic and static components [18] were recruited (n = 34). Highly trained athletes in competition or training period with definite ARVC were also enrolled (n = 8). Ethical approval was obtained from the Central Ethics Committee of Hungary and has been performed in accordance with the ethical standards laid down in the 1964 Declaration of Helsinki and its later amendments. All subjects gave their informed consent prior to their inclusion in the study.

### CMR examination

CMR examinations were conducted on a 1.5 T MR scanner (Achieva, Philips Medical Systems) with a 5-channel cardiac coil. Locaizing scans were followed by breath-hold cine imaging in transversal planes. Retrospectively-gated, balanced steady-state free precession (bSSFP) segmented cine images were acquired in 2-chamber, 4-chamber and LV outflow tract views. Short-axis images with full coverage of the left and right ventricle, and RV outflow tract images were obtained with a temporal resolution ≤ 40 ms, with a mean echo time/repetition time/flip angle: 1.35 ms/2.7 ms/60°, respectively. 30 phases per cardiac cycle were acquired with one or two slices per 10–14 s breath-hold depending on the patients/individuals breath-hold capacity. Image domain based parallel imaging (SENSE) was used in case of short-axis movies (acceleration factor 2.0). Spatial resolution regardless of the field-of view (350 mm on average adapted to body size) with a mean acquisition pixel size of 1.6 mm × 1.6 mm, slice thickness was 8 mm with no interslice gap.

Late gadolinium enhancement (LGE) imaging was performed if patients gave their informed consent (82.4% of healthy athletes and 94.1% of ARVC patients, 100% of athletes with ARVC). During an inspiratory breath-hold, a bolus of gadobutrol (0.15 mmol/kg) was injected at a rate of 2–3 ml/s through antecubital intravenous line. Contrast-enhanced images were acquired using a segmented inversion recovery sequence with additional phase sensitive reconstructions in long- and short-axis views (slice thickness 8 mm with no interslice gap, mean echo time 2.2/repetition time 4.6/flip angle: 15) 10–20 min after contrast administration. Parallel imaging is not utilized in LGE images. The inversion-time was adjusted to provide optimal suppression of apparent normal myocardium.

### Image analysis

All images were evaluated with Medis Suite (QMass and QStrain) Software (Medis Medical Imaging Systems, version 3.0, Leiden, The Netherlands). Endocardial and epicardial contour detection was performed by a blinded expert observer manually on short axis cine images in end-systolic and end-diastolic phases. Quantification of the left and right ventricular ejection fraction (LVEF, RVEF), end-systolic volume (LVESV, RVESV), end-diastolic volume (LVEDV, RVEDV), stroke volume (LVSV, RVSV) and myocardial mass (LVM, RVM) were performed. Left and right ventricular volumes and masses were standardized to body surface area (BSA)—LVESVi, RVESVi, LVEDVi, RVEDVi, LVSVi, RVSVi, LVMi and RVMi. Regional right ventricular akinesis, dyskinesis, dyssynchrony were qualitatively assessed. Global LV and RV strain analysis was performed based on cine images after manual contouring of the endocardial borders. Additionally, regional strain analysis for the right ventricular free wall was performed based on RV endocardial contours on 4CH-view, peak systolic longitudinal strain and strain rate values of the basal, midventricular and apical free wall were established. Average and minimal values of the measured regional strain and strain rate values were also determined.

### Statistical analysis

Continuous variables were reported as mean and standard deviation, and categorical variables as frequency and percentages. Between-group comparisons were performed with the unpaired Student’s *t* test, or Mann–Whitney U-test where appropriate. Diagnostic accuracy of CMR parameters was evaluated using receiver operating characteristic (ROC) curve analysis with ≥ 4 Task Force Score (major criteria = 2 points, minor criteria = 1 point). For area under the curve (AUC), a value of 0.9–1.0 was considered excellent, 0.75–0.9 good, 0.6–0.75 moderate and 0.5–0.6 poor. Optimal cut-off values for ARVC patient vs. athlete classification were derived from ROC curves coordinates by maximizing the proportion of subjects correctly classified. A p-value of ≤ 0.05 was considered statistically significant. Statistical analysis was performed with MedCalc software (version 17.9).

## Results

### Baseline characteristics

#### Healthy athletes

Thirty-four highly trained healthy athletes with no signs of cardiovascular disease and with a minimum of 15 h of training per week for at least 5 years performing sports with high dynamic and static components (31.8 ± 6.1 years, 22 male, 18.6 ± 2.2 training h/week) were recruited, including canoe and kayakers (n = 17), rowers (n = 5), boxers (n = 5), triathletes (n = 4) and cyclists (n = 3). None of the healthy athletes had positive family history of sudden cardiac death or ARVC, all of them were asymptomatic. None of the healthy athletes had any ECG abnormality suggesting structural heart disease, none of them showed T-wave inversion in more than 1 continuous leads, Q wave inversion or ST-depression. 81% of athletes showed J-point elevation, 19% Sokolow or Cornell index positivity for left ventricular hypertrophy. None of them showed regional right ventricular wall motion abnormality or late gadolinium enhancement (LGE). RVEDVi was in the proposed range of the major TFC (> 110 ml/m^2^ in males, > 100 ml/m^2^ in females) in all healthy male athletes and 83.3% of healthy female athletes. RVEDVi of the remaining female athletes were in the proposed range of the minor TFC (> 90 ml/m^2^ in females). None of the athletes showed RVEF ≤ 45%, RVEF between 45 and 50% was observed in 5 cases (14.7%, three male and two female athletes).

#### ARVC patients

**Fig. 1:**
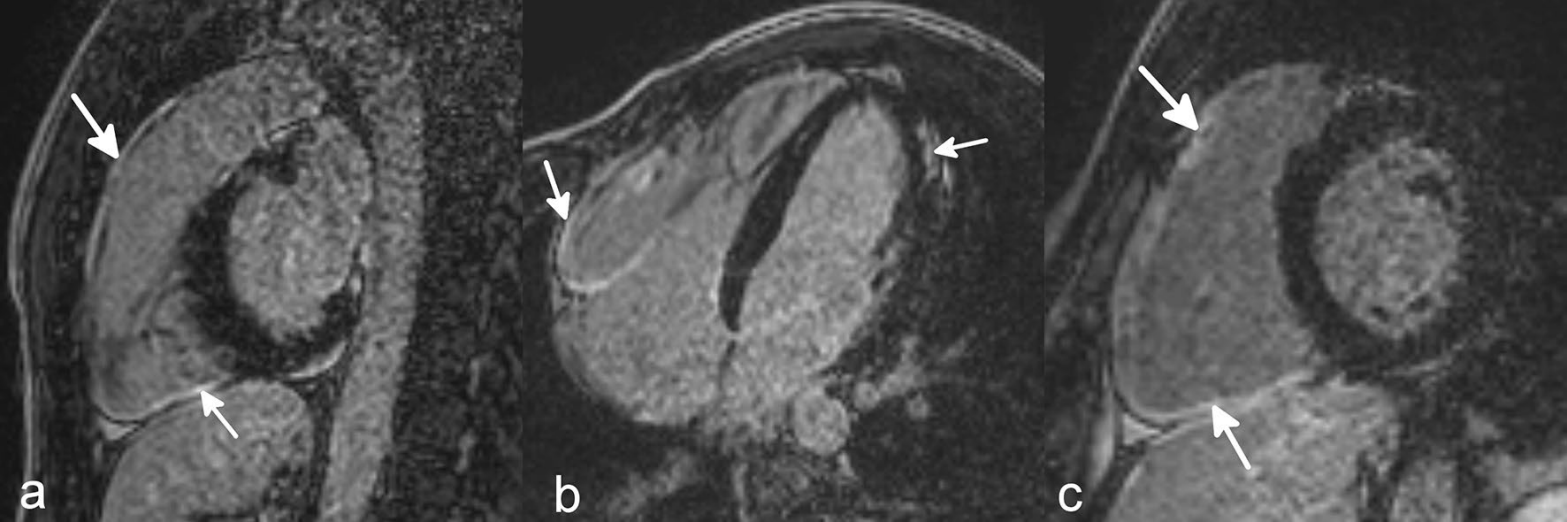
LGE images of an athlete with biventricular ARVC

Diagnosis of ARVC was based on current TFC [4]. Thirty-four non-athlete ARVC patients were enrolled (40.5 ± 13.4 years, 22 male). Positive family history for sudden cardiac death or ARVC was present in 18% of the ARVC patients, 59% had recorded sustained ventricular tachycardia (VT) or ventricular fibrillation (VF), and aborted sudden cardiac death was reported in 12%. Average Task Force score (major = 2 points, minor = 1 point) was 4.9 points. Biventricular involvement was observed in 71%. LGE was present in 69%, in 14 patients only in the LV, in two patients only in the RV, and 6 patients demonstrated biventricular LGE (Fig. 1).

#### Athletes with ARVC

**Fig. 2:**
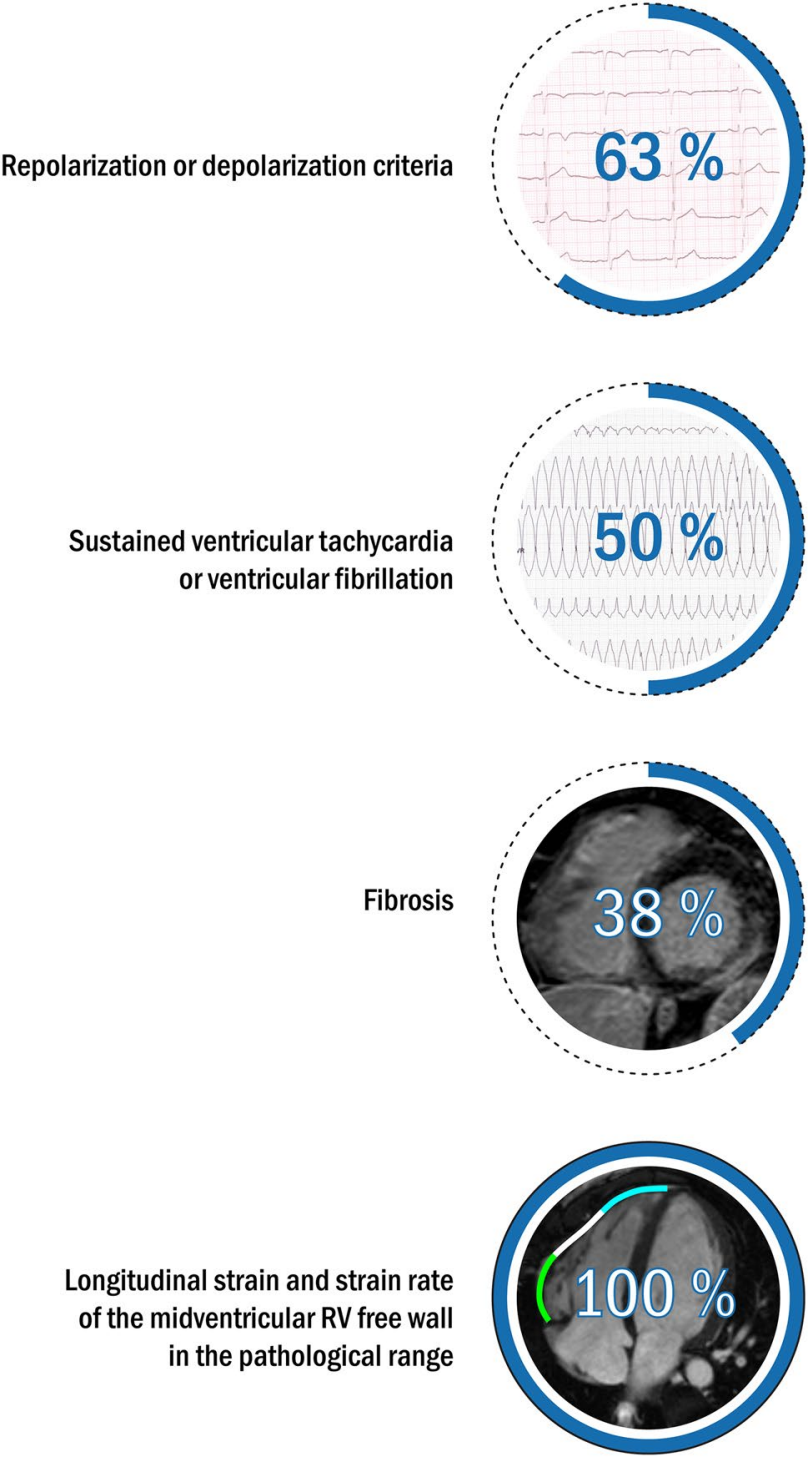
Clinical characteristics of athletes with ARVC including pathological ECG findings, arrhythmias and myocardial fibrosis. Midventricular RV longitudinal strain and strain rate were in the pathological range based on our cut-off values in all of the athletes with ARVC

In eight additional active athletes (18.9 ± 4.6 training h/week, triathletes n = 3, cyclists n = 2, swimmers n = 2, football player n = 1) the comprehensive investigation including resting ECG, ambulatory ECG monitoring, echocardiography, CMR and patient history confirmed the diagnosis (Fig. 2). In three cases, CMR examination was repeated after training cessation for 2–3 months. Successful deconditioning effect was observed on LV values (LVEDVi: 127.4 ± 5.4 ml/m^2^ vs. 109 ± 10.6 ml/m^2^; LVMi: 93.4 ± 16.7 vs. 73.5 ± 9.4), but only blunted deconditioning effect was observed on RV values, RVEDVi remained in the pathological range (169.7 ± 10.5 ml/m^2^ vs. 137.3 ± 17.1 ml/m^2^). Four athletes had documented sustained VT or VF, and aborted sudden cardiac death was reported in two cases. None of them had positive family history for ARVC or SCD. Two athletes did not fulfil any repolarization or depolarization criteria, TWI in lead v1–v2 and in v1–v3 leads was observed in one and four athletes, respectively. One patient had complete right bundle branch block. Epsilon wave was observed in 3 patients. Average Task Force score was 5.4 points (Table 1). Biventricular involvement was confirmed in three patients (defined by left ventricular LGE and/or LVEF < 50). Three patients showed LGE: two of them biventricular and one solely left ventricular LGE. All athletes with definite diagnosis of ARVC were disqualified from competitive sport.

**Table 1:** Baseline characteristics and task force criteria of healthy athletes, sedentary ARVC patients and athletes with ARVC

	Athletes (n = 34)	ARVC (n = 34)	Athletes with ARVC (n = 8)
Task force criteria:			
Global or regional dysfunction and structural alterations			
Major	0/34	30/34	8/8
Minor	0/34	4/34	0/8
Tissue characterization of wall			
Major	–	2/3	–
Minor	–	1/3	–
Repolarization abnormalities			
Major	0/34	22/34	4/8
Minor	0/34	1/34	1/8
Depolarization abnormalities			
Major	0/34	2/34	2/8
Minor	0/34	6/34	2/8
Arrhythmias			
Major	0/34	7/34	4/8
Minor	0/34	21/34	3/8
Family history			
Major	0/34	3/33	0/8
Minor	0/34	3/33	0/8
TF points	0.0 ± 0.0	4.9 ± 0.7	5.4 ± 1.2

TF points: major = 2, minor = 1 points

### Characteristics of patient and subject groups—comparison of CMR parameters between healthy athletes and ARVC patients

Healthy athletes showed higher LVEDVi, LVSVi, LVMi, RVSVi and RVMi than non-athlete ARVC patients, no significant difference was found between athletes and non-athlete ARVC patients regarding the RVEDVi. Both RVEF and LVEF were significantly lower in ARVC patients compared to healthy athletes (Table 2).

**Table 2:** Baseline characteristics and conventional left and right ventricular CMR parameters in healthy athletes, ARVC patients and athletes with ARVC

	Athletes (n = 34) Mean ± SD	ARVC (n = 34) Mean ± SD	Athletes with ARVC (n = 8) Mean ± SD
Age (years)	31.8 ± 7.7 [28.7–34.0]	40.5 ± 16.3* [32.5–44.3]	27.6 ± 3.3 [24.4–30.7]
BSA (m^2^)	1.94 ± 0.19 [1.87–2.06]	1.88 ± 0.21 [1.78–2.00]	1.86 ± 0.13 [1.70–2.02]
Male (%)	65	65	87.5
Heart rate (bpm)	55.0 ± 7.6 [50.0–60.0]	63.0 ± 7.4*** [61.0–66.2]	58.5 ± 8.8 [46.0–69.1]
LVEF (%)	57.6 ± 4.7 [54.6–60.5]	52.0 ± 11.2* [46.9–57.8]	60.1 ± 3.1 [56.0–64.5]
LVESVi (ml/m^2^)	50.3 ± 10.9 [43.2–53.4]	50.9 ± 20.0 [40.0–50.9]	50.2 ± 8.3 [41.0–61.8]
LVEDVi (ml/m^2^)	117.8 ± 17.3 [107.8–122.6]	107.2 ± 22.5* [93.5–117.0]	121.1 ± 17.4 [95.1–142.2]
LVSVi (ml/m^2^)	67.6 ± 9.4 [63.4–71.0]	54.0 ± 13.1*** [50.1–58.7]	72.6 ± 10.9 [60.5–83.7]
LVMi (g/m^2^)	82.9 ± 16.1 [76.2–89.0]	58.2 ± 12.8*** [51.1–61.4]	87.2 ± 16.3 [68.7–109.8]
RVEF (%)	55.7 ± 4.6 [54.2–57.3]	40.5 ± 13.6*** [34.5–46.6]	47.5 ± 3.8 [42.4–52.2]
RVESVi (ml/m^2^)	55.6 ± 11.9 [49.0–56.2]	88.3 ± 45.1*** [62.4–89.4]	76.2 ± 10.8 [62.7–97.4]
RVEDVi (ml/m^2^)	123.6 ± 17.0 [111.7–130.4]	142.7 ± 47.5 [117.4–138.5]	146.1 ± 25.7 [114.0–179.0]
RVSVi (ml/m^2^)	68.6 ± 9.1 [64.0–72.7]	52.7 ± 17.5*** [50.7–63.8]	70.0 ± 15.1 [49.5–89.2]
RVMi (g/m^2^)	21.8 ± 6.2 [18.1–25.2]	18.7 ± 4.8 [14.7–20.4]	22.3 ± 2.3 [19.8–24.4]

*BSA* body surface area, *LV* left ventricular, *RV* right ventricular, *EF* ejection fraction, *ESVi* end-systolic volume index, *EDVi* end-diastolic volume index, *SVi* stroke volume index, *Mi* mass index

Values are expressed in mean ± SD [95% Confidence Interval]

*p < 0.05; **p < 0.01; ***p < 0.001, Significant difference between sedentary ARVC patients and healthy athletes

RV global longitudinal strain (RV GLS) was decreased in the ARVC group compared to athletes. Regional longitudinal strain and strain rates of the RV free wall are presented in Table 3. RV mid strain, RV mid strain rate, and average and minimal values of the measured regional strain and strain rate values showed significant difference between the two groups (Table 3).

**Table 3:** Global and regional strain and strain rate values of healthy athletes, ARVC patients and athletes with ARVC

	Athletes (n = 34) Mean ± SD	ARVC (n = 34) Mean ± SD	Athletes with ARVC (n = 8) Mean ± SD
Global left and right ventricular strain			
LV GLS	− 22.0 ± 3.4 [− 23.4 to − 20.6]	− 20.4 ± 5.0 [− 22.5 to − 18.2]	− 24.9 ± 1.4 [− 27.6 to − 23.2]
LV GCS	− 30.5 ± 6.0 [− 32.5 to − 27.1]	− 26.3 ± 7.8* [− 30.9 to − 21.9]	− 35.6 ± 5.7 [− 41.4 to − 29.3]
LV GRS	57.6 ± 14.5 [49.3–58.5]	49.8 ± 49.9 [40.7–58.3]	71.4 ± 11.2 [58.0–87.2]
RV GLS	− 25.6 ± 3.9 [− 27.7 to − 23.7]	− 20.4 ± 7.6** [− 24.1 to − 16.7]	− 21.3 ± 3.9 [− 26.0 to − 17.0]
Regional longitudinal strain and strain rate values of the RV free wall			
RV basal strain	− 34.5 ± 8.6 [− 39.3 to − 32.1]	− 30.9 ± 11.8 [− 35.1 to − 24.6]	− 35.6 ± 8.3 [− 43.7 to − 28.7]
RV mid strain	− 31.5 ± 10.2 [− 36.4 to − 27.8]	− 20.0 ± 13.4*** [− 23.4 to − 12.0]	− 21.5 ± 4.9 [− 25.3 to − 14.6]
RV apical strain	− 30.9 ± 8.0 [− 35.8 to − 27.4]	− 24.6 ± 11.6** [− 28.2 to − 19.6]	− 19.7 ± 11.1 [− 29.1 to − 8.2]
RV average strain	− 32.3 ± 5.0 [− 34.1 to − 31.2]	− 25.1 ± 9.3** [− 27.4 to − 22.7]	− 25.6 ± 3.0 [− 29.7 to − 23.2]
RV min strain	− 24.0 ± 7.2 [− 27.4 to − 22.7]	− 15.2 ± 9.0*** [− 17.1 to − 10.4]	− 15.4 ± 7.1 [− 23.8 to − 8.2]
RV basal strain rate	− 1.74 ± 0.59 [− 2.00 to − 1.40]	− 1.52 ± 0.97* [− 1.70 to − 0.90]	− 1.61 ± 0.63 [− 2.40 to − 0.94]
RV mid strain rate	− 1.37 ± 0.56 [− 1.70 to − 1.08]	− 1.04 ± 0.68** [− 1.20 to − 0.70]	− 1.05 ± 0.22 [− 1.22 to − 0.78]
RV apical strain rate	− 1.33 ± 0.40 [− 1.50 to − 1.10]	− 1.17 ± 0.56 [− 1.32 to − 0.80]	− 0.96 ± 0.34 [− 1.32 to − 0.71]
RV average strain rate	− 1.48 ± 0.38 [− 1.53 to − 1.33]	− 1.24 ± 0.59* [− 1.43 to − 0.86]	− 1.21 ± 0.28 [− 1.49 to − 0.92]
RV min strain rate	− 1.09 ± 0.31 [− 1.20 to − 0.90]	− 0.79 ± 0.32** [− 0.92 to − 0.60]	− 0.80 ± 0.24 [− 1.02 to − 0.62]

Values are expressed in mean ± SD [95% Confidence Interval]

*GLS* global longitudinal strain, *GCS* global circumferential strain, *GRS* global regional strain

*p < 0.05; **p < 0.01; ***p < 0.001, Significant difference between sedentary ARVC patients and healthy athletes

### Diagnostic accuracy of CMR parameters and feature tracking based deformation imaging to differentiate ARVC and athlete’s heart

Establishing AUC values for the CMR parameters RVEF showed good accuracy (AUC = 0.830), whereas RVEDVi failed as a discriminator between ARVC and athlete’s heart (AUC = 0.599). Cut-off value for RVEF ≤ 45.8 discriminates between ARVC and athlete’s heart with a sensitivity of 68% and a specificity of 100%. RV mid strain, average and minimum of the measured regional strain values demonstrated good discrimination between athlete’s heart and ARVC. We investigated whether establishing gender-specific cut-off values may influence the diagnostic accuracy, but comparing male and female ROC curves for CMR parameters with the highest diagnostic accuracy (RVEF, RV mid strain, RV average strain, RV min strain) showed no significant difference. These results suggests that gender-specific differences regarding these parameters are negligible, so we present cut-off values for both males and females. Cut-off values for global and regional strain values are presented in Table 4.

**Table 4:** Area under the ROC curves and cut-off values for optimised sensitivity and specificity

	Cut-off value	AUC	Sensitivity	Specificity	p
CMR task force criteria					
RVEF	≤ 45.8	**0.830**	67.65	100.00	0.0001
RVEDVi	> 150.8	0.599	29.41	94.12	NS
Global left and right ventricular strain values					
LV GLS	> − 17.7	0.596	32.35	94.12	NS
LV GCS	> − 22.5	0.643	38.24	100.00	0.0386
LV GRS	≤ 41.8	0.607	41.18	94.12	NS
RV GLS	> − 20.1	0.726	50.00	97.06	0.0004
Regional strain values of the RV free wall					
RV basal strain	< − 35.8	0.634	70.59	58.82	NS
RV mid strain	> − 25.6	**0.767**	70.59	82.35	0.0001
RV apical strain	> − 23.9	0.694	55.88	85.29	0.0042
RV average strain	> − 29.4	**0.772**	73.53	76.47	0.0001
RV min strain	> − 18.1	**0.786**	70.59	85.29	0.0001
RV basal strain rate	> − 1.3	0.665	58.82	85.29	0.0195
RV mid strain rate	> − 1.4	0.686	82.35	50.00	0.0045
RV apical strain rate	> − 0.9	0.606	38.24	91.18	NS
RV average strain rate	> − 1.13	0.665	52.94	88.24	0.0175
RV min strain rate	> 0.8	0.702	55.88	82.35	0.0014

Parameters showing good accuracy appears in bold

**Fig. 3:**
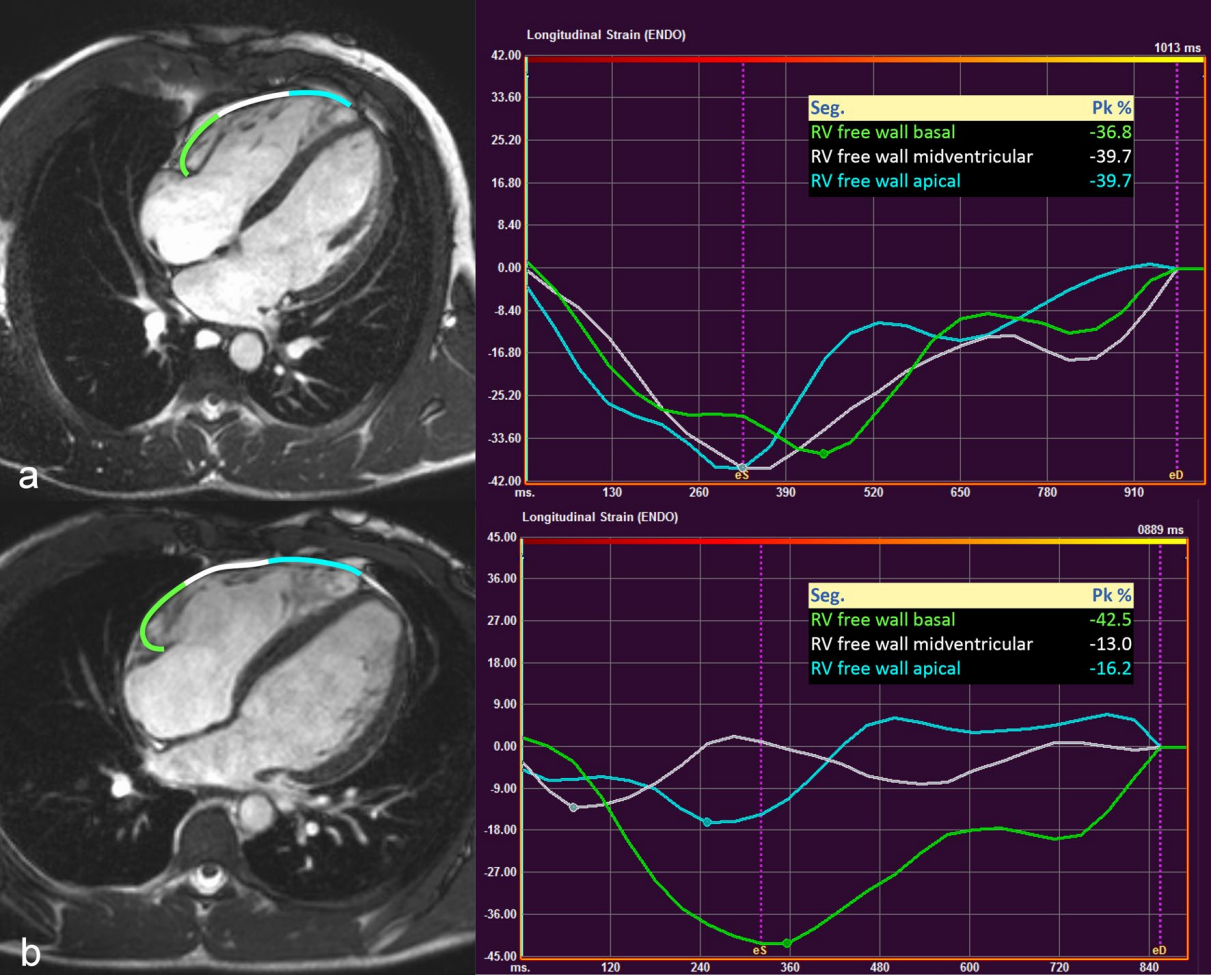
Regional free RV wall strain analysis of a healthy athlete (**a**) and a highly trained athlete with ARVC (**b**). Strain curves of the athlete with ARVC represent regional RV dyssynchrony and decreased longitudinal strain of the midventricular (white) and apical (blue) RV free wall. Segments of the RV free wall and regional strain values of these segments are presented using the same colour

We tested the established cut-off values for CMR parameters in the group of highly trained athletes with diagnosed ARVC. Applying the established cut-off values, RVEF was in the pathological range in only 3 athletes with ARVC. Half of the athletes with ARVC showed normal RV GLS. Regional longitudinal strain and strain rate of the RV mid free wall were in the pathological range among all 8 athletes with ARVC (Fig. 3).

## Discussion

Highly trained athletes participating in our study reached the major Task Force criteria on RV dilatation in almost 95%. Based on these findings, RVEDVi failed as a discriminator, highlighting the difficulties of differentiation between ARVC and athlete’s heart using the modified Task Force criteria. Although none of the athletes in this population showed RVEF under 45%, previous CMR studies regarding reference RV values of athletes showed that RVEF between 40 and 45% could be observed in 5% of elite athletes, and pronounced RV dilatation is a typical feature in healthy athletes, even with only moderate training [19, 20].

Although CMR is the gold standard method to evaluate RV function and volumes, subjective assessment of right ventricular wall thinning and wall motion abnormalities in the clinical routine represents the Achilles’ heel of CMR. Deformation imaging using feature tracking and novel methods such as ventricular shape analysis [21] may have an important added value in the objective assessment of right ventricular wall motion abnormalities. Based on the expert consensus document of the European Association of Cardiovascular Imaging (EACVI) regarding multi-modality imaging in arrhythmogenic right ventricular cardiomyopathy, deformation strain imaging is recommended in patients with suspected ARVC [22]. RV deformation imaging using two-dimensional speckle tracking echocardiography, although currently available, is still challenging [23]. Based on recent data, global and regional right ventricular strain values of overt ARVC patients are decreased compared to healthy control subjects [5–8]. Moreover, multisoftware feasibility study of Bourfiss et al. proved excellent intra- and inter-observer reproducibility for the right ventricular regional strain values established using Medis QStrain software [5].

One advantage of CMR based strain analysis using feature tracking is the opportunity for retrospective analysis because no additional image acquisition is required. CMR based strain analysis moreover provides better tissue contrast and superior definition of the endocardial layer compared to speckle-tracking echocardiography, on the other hand speckle-tracking echocardiography has better spatial and temporal resolution. Average temporal and spatial resolutions expected from CMR-FT are: 25–35 phases/cardiac cycle depending on heart rate (eg, 30–40 ms at a heart rate of 60 bpm) and 1–2 × 1–2 mm in plane resolution with a slice thickness of 6–8 mm [24]. Technical improvements in the future may further optimize spatial and temporal resolution contributing to more precise strain analysis.

Although several echocardiographic studies have investigated the morphologic right ventricular adaptation of elite athletes [25], limited published data are available about CMR derived upper normal limit or cut-off values to differentiate athlete’s heart and ARVC [14, 19, 20]. This is the first study to provide strain values on healthy athletes using feature tracking and compare it to ARVC patients’ strain parameters.

Because of the pronounced RV adaptation observed in healthy highly trained athletes, diagnosing ARVC in elite athletes is more challenging than in sedentary individuals. Our findings imply that the phenotype of sedentary and athletic ARVC patients differ. We are the first to report detailed functional and morphological characteristics, and CMR based strain values of an athlete cohort with definite ARVC in training or competition period. According to literature data ejection fraction may stay in normal range especially in athletes with structural heart disease, but literature data imply that decreased strain values could indicate decreased function earlier than ejection fraction [26]. Based on our small cohort, highly trained athletes with definite ARVC may show good RVEF and normal global strain values, while regional strain and strain rate values are decreased.

Regional strain and strain rate of the right ventricular mid free wall are valuable discriminators even in patients with preserved RVEF and normal RV GLS. This fact highlights the significance of reporting CMR based strain parameters for differentiating athlete’s heart and ARVC. Due to the moderate sensitivity our cut-off values confirms that CMR is not an appropriate first line screening method, good or excellent specificity enhances its role to prevent unnecessary disqualification because of overdiagnosing ARVC.

The major limitation of our study is the single-center nature and the limited number of participants. Additionally, our ARVC patients represent an older population compared to our athletes, although age differences may not necessarily influence our results—there have been contradictory data originating from the studies on the effect of aging on strain values [27, 28]. Another limitation of our study are the gender differences between the athletes with ARVC and the other two groups. Genetic mutation screening was not routinely performed in our patient population.

## Conclusion

Our study highlights that RV dilatation in healthy endurance athletes with regular and intensive training may reach the proposed Task Force criteria in almost 95%. Therefore, elevated RVEDVi is an insufficient criterion for morphological diagnosis of ARVC. Besides establishing RVEF using CMR, RV strain analysis can provide an important tool to diagnose ARVC and distinguish it from athlete’s heart. CMR based regional strain and strain rate values may help to identify ARVC even in highly trained athletes with preserved RVEF and normal RV GLS.

## References

[1] D’Ascenzi F, Pisicchio C, Caselli S, Di Paolo FM, Spataro A, Pelliccia A (2017). RV remodeling in olympic athletes. JACC Cardiovasc Imaging.

[2] Corrado D, Basso C, Rizzoli G, Schiavon M, Thiene G (2003). Does sports activity enhance the risk of sudden death in adolescents and young adults?. J Am Coll Cardiol.

[3] Thiene G, Nava A, Corrado D, Rossi L, Pennelli N (1988). Right ventricular cardiomyopathy and sudden death in young people. N Engl J Med.

[4] Marcus FI, McKenna WJ, Sherrill D, Basso C, Bauce B, Bluemke DA (2010). Diagnosis of arrhythmogenic right ventricular cardiomyopathy/dysplasia: proposed modification of the task force criteria. Eur Heart J.

[5] Bourfiss M, Vigneault DM, Aliyari Ghasebeh M, Murray B, James CA, Tichnell C (2017). Feature tracking CMR reveals abnormal strain in preclinical arrhythmogenic right ventricular dysplasia/cardiomyopathy: a multisoftware feasibility and clinical implementation study. J Cardiovasc Magn Reson.

[6] Prati G, Vitrella G, Allocca G, Muser D, Buttignoni SC, Piccoli G (2015). Right ventricular strain and dyssynchrony assessment in arrhythmogenic right ventricular cardiomyopathy: cardiac magnetic resonance feature-tracking study. Circ Cardiovasc Imaging.

[7] Heermann P, Hedderich DM, Paul M, Schulke C, Kroeger JR, Baessler B (2014). Biventricular myocardial strain analysis in patients with arrhythmogenic right ventricular cardiomyopathy (ARVC) using cardiovascular magnetic resonance feature tracking. J Cardiovasc Magn Reson.

[8] Vigneault DM, te Riele AS, James CA, Zimmerman SL, Selwaness M, Murray B (2016). Right ventricular strain by MR quantitatively identifies regional dysfunction in patients with arrhythmogenic right ventricular cardiomyopathy. J Magn Reson Imaging.

[9] Lie OH, Dejgaard LA, Saberniak J, Rootwelt C, Stokke MK, Edvardsen T (2018). Harmful effects of exercise intensity and exercise duration in patients with arrhythmogenic cardiomyopathy. JACC Clin Electrophysiol.

[10] Wang W, Orgeron G, Tichnell C, Murray B, Crosson J, Monfredi O (2018). Impact of exercise restriction on arrhythmic risk among patients with arrhythmogenic right ventricular cardiomyopathy. J Am Heart Assoc.

[11] Mont L, Pelliccia A, Sharma S, Biffi A, Borjesson M, Brugada Terradellas J (2017). Pre-participation cardiovascular evaluation for athletic participants to prevent sudden death: position paper from the EHRA and the EACPR, branches of the ESC. Endorsed by APHRS, HRS, and SOLAECE. Eur J Prev Cardiol.

[12] Ruwald AC, Marcus F, Estes NA, Link M, McNitt S, Polonsky B (2015). Association of competitive and recreational sport participation with cardiac events in patients with arrhythmogenic right ventricular cardiomyopathy: results from the North American multidisciplinary study of arrhythmogenic right ventricular cardiomyopathy. Eur Heart J.

[13] Saberniak J, Hasselberg NE, Borgquist R, Platonov PG, Sarvari SI, Smith HJ (2014). Vigorous physical activity impairs myocardial function in patients with arrhythmogenic right ventricular cardiomyopathy and in mutation positive family members. Eur J Heart Fail.

[14] Luijkx T, Velthuis BK, Prakken NH, Cox MG, Bots ML, Mali WP (2012). Impact of revised task force criteria: distinguishing the athlete’s heart from ARVC/D using cardiac magnetic resonance imaging. Eur J Prev Cardiol.

[15] Biffi A, Pelliccia A, Verdile L, Fernando F, Spataro A, Caselli S (2002). Long-term clinical significance of frequent and complex ventricular tachyarrhythmias in trained athletes. J Am Coll Cardiol.

[16] Choung HYG, Vyas M, Jacoby D, West B (2017). Arrhythmogenic right ventricular cardiomyopathy (ARVC) in a young female athlete at 36 weeks gestation: a case report. Pathol Res Pract.

[17] Hedley JS, Al Mheid I, Alikhani Z, Pernetz MA, Kim JH (2017). Arrhythmogenic right ventricular cardiomyopathy in an endurance athlete presenting with ventricular tachycardia and normal right ventricular function. Tex Heart Inst J.

[18] Levine BD, Baggish AL, Kovacs RJ, Link MS, Maron MS, Mitchell JH (2015). Eligibility and disqualification recommendations for competitive athletes with cardiovascular abnormalities: task force 1: classification of sports: dynamic, static, and impact: a scientific statement from the American heart association and American college of cardiology. J Am Coll Cardiol.

[19] Prakken NH, Velthuis BK, Teske AJ, Mosterd A, Mali WP, Cramer MJ (2010). Cardiac MRI reference values for athletes and nonathletes corrected for body surface area, training hours/week and sex. Eur J Cardiovasc Prev Rehabil.

[20] Bohm P, Schneider G, Linneweber L, Rentzsch A, Kramer N, Abdul-Khaliq H (2016). Right and left ventricular function and mass in male elite master athletes: a controlled contrast-enhanced cardiovascular magnetic resonance study. Circulation.

[21] McLeod K, Wall S, Leren IS, Saberniak J, Haugaa KH (2016). Ventricular structure in ARVC: going beyond volumes as a measure of risk. J Cardiovasc Magn Reson.

[22] Haugaa KH, Basso C, Badano LP, Bucciarelli-Ducci C, Cardim N, Gaemperli O (2017). Comprehensive multi-modality imaging approach in arrhythmogenic cardiomyopathy—an expert consensus document of the European association of cardiovascular imaging. Eur Heart J Cardiovasc Imaging.

[23] Badano LP, Kolias TJ, Muraru D, Abraham TP, Aurigemma G, Edvardsen T (2018). Standardization of left atrial, right ventricular, and right atrial deformation imaging using two-dimensional speckle tracking echocardiography: a consensus document of the EACVI/ASE/Industry task force to standardize deformation imaging. Eur Heart J Cardiovasc Imaging.

[24] Schuster A, Hor KN, Kowallick JT, Beerbaum P, Kutty S (2016). Cardiovascular magnetic resonance myocardial feature tracking: concepts and clinical applications. Circ Cardiovasc Imaging.

[25] Oxborough D, Sharma S, Shave R, Whyte G, Birch K, Artis N (2012). The right ventricle of the endurance athlete: the relationship between morphology and deformation. J Am Soc Echocardiogr.

[26] Teske AJ, Cox MG, Te Riele AS, De Boeck BW, Doevendans PA, Hauer RN (2012). Early detection of regional functional abnormalities in asymptomatic ARVD/C gene carriers. J Am Soc Echocardiogr.

[27] Liu B, Dardeer AM, Moody WE, Edwards NC, Hudsmith LE, Steeds RP (2018). Normal values for myocardial deformation within the right heart measured by feature-tracking cardiovascular magnetic resonance imaging. Int J Cardiol.

[28] Taylor RJ, Moody WE, Umar F, Edwards NC, Taylor TJ, Stegemann B (2015). Myocardial strain measurement with feature-tracking cardiovascular magnetic resonance: normal values. Eur Heart J Cardiovasc Imaging.

